# Causal associations between *Helicobacter Pylori* infection and the risk and symptoms of Parkinson’s disease: a Mendelian randomization study

**DOI:** 10.3389/fimmu.2024.1412157

**Published:** 2024-08-06

**Authors:** Xin Wang, Deming Jiang, Xiao Zhang, Ran Wang, Fengyi Yang, Chunrong Xie

**Affiliations:** ^1^ Department of Neurology, Beijing Huairou Hospital of Traditional Chinese Medicine, Beijing, China; ^2^ Department of Neurology, Xuanwu Hospital, Capital Medical University, Beijing, China

**Keywords:** Mendelian randomization, Parkinson’s disease, *Helicobacter pylori*, motor subtype, symptom

## Abstract

**Background:**

Increasing evidence suggests an association between *Helicobacter pylori* (HP) infection and Parkinson’s disease (PD) and its clinical manifestations, but the causal relationship remain largely unknown.

**Objective:**

To investigate the causal relationship between HP infection and PD risk, PD symptoms, and secondary parkinsonism, we conducted two-sample Mendelian randomization (MR).

**Methods:**

We obtained summary data from genome-wide association studies for seven different antibodies specific to HP proteins and five PD-related phenotypes. The inverse-variance weighted (IVW), weighted median, weighted mode, and MR-Egger methods were used to assess the causal relationships. Sensitivity analyses were performed to examine the stability of the MR results and reverse MR analysis was conducted to evaluate the presence of reverse causality.

**Results:**

Genetically predicted HP antibodies were not causally associated with an increased risk of PD. However, HP cytotoxin-associated gene-A (CagA) and outer membrane protein (OMP) antibody level were causally associated with PD motor subtype (tremor to postural instability/gait difficulty score ratio; *β* = -0.16 and 0.46, *P* = 0.002 and 0.048, respectively). HP vacuolating cytotoxin-A (VacA) antibody level was causally associated with an increased risk of PD dementia [odds ratio (OR) = 1.93, *P* = 0.040]. Additionally, HP OMP antibody level was identified as a risk factor for drug-induced secondary parkinsonism (OR = 2.08, *P* = 0.033). These results were stable, showed no evidence of heterogeneity or directional pleiotropy, and no evidence of a reverse causal relationship.

**Conclusions:**

Our findings indicate that HP infection does not increase the risk of PD, but contributes to PD motor and cognitive symptoms. Different types of HP antibodies affect different symptoms of PD. Eradication of HP infection may help modulate and improve symptoms in PD patients.

## 1 Introduction

Parkinson’s disease (PD) is a prevalent neurodegenerative disorder, ranking second only to Alzheimer’s disease in terms of disease burden (1). It is estimated that by 2040, the global number of patients with PD will exceed 14 million (2). The clinical manifestations of PD can be classified into motor symptoms and non-motor symptoms. Motor symptoms primarily involve bradykinesia, tremors, gait difficulty, and postural instability, while non-motor symptoms mainly include depression, constipation, sleep disorders, and cognitive impairments (3). The etiology of PD is not yet fully understood. The current understanding is that the pathogenesis of PD is primarily associated with the deposition of unidentified α-synuclein in the brain and the loss of nigrostriatal dopamine neurons (4). Recently, the role of microorganisms in the pathogenesis of PD has gained significant attention (5–7). Notably, increasing evidence suggests a close association between *Helicobacter pylori* (HP) infection and PD (8).

*Helicobacter pylori* is a Gram-negative, microaerophilic, and flagellated bacterium that primarily colonizes the human gastric mucosa, causing conditions such as gastric ulcers and duodenal ulcers (8). After infecting the human body, HP can secrete various toxins and then induce the production of various antibodies in host, including anti-H. pylori IgG, cytotoxin-associated gene-A (CagA), catalase, chaperonin GroEL (GroEL), outer membrane protein (OMP), urease subunit-A (UreA), and vacuolating cytotoxin-A (VacA) antibodies (9–11). The specific toxins and antibodies generated depend on the strain of HP infecting the host and its genetic profile (10). Among them, CagA and VacA toxins can further induce systemic inflammation and neuroinflammation (12). The mechanisms through which HP infection is associated with PD may include the production of HP-related toxins, stimulation of pro-inflammatory cytokines, interference with the effectiveness of PD medications, and small intestinal bacterial overgrowth (8). Previous studies have shown that the prevalence of HP infection in patients with PD is approximately 1.6 times higher than in control groups (13). Our previous umbrella review indicates that patients with PD can have multiple microbial infections, with the highest level of evidence related to HP infection (14). However, a systematic review has shown that the prevalence of HP infection in PD ranges from 37% to 59%, similar to the general population (15). Furthermore, HP infection is also associated with the clinical manifestations and severity of PD (16, 17). Meta-analysis shows that PD patients with HP infection have more severe motor symptoms and poorer response to medication (16). However, it is worth noting that the results of intervention studies are inconsistent. Some small-sample studies suggest that HP eradication therapy can effectively improve the clinical symptoms of PD, particularly bradykinesia and stride length (18). However, a larger randomized controlled trial of HP eradication in PD suggests that eradicating HP does not improve the clinical outcomes of PD, including both motor and non-motor symptoms (19). Based on current research findings, the direct link between HP infection and PD, as well as its impact on the clinical symptoms of PD, remains inconclusive. However, establishing a definitive causal relationship is crucial for developing preventive measures or treatment strategies for PD by eradicating HP.

Mendelian randomization (MR) is a causal inference epidemiological analysis method. It integrates summary data from genome-wide association studies (GWAS) to determine the causal relationship between exposure and outcome (20). Genetic variants, specifically single-nucleotide polymorphisms (SNPs), are used as instrumental variables (IVs) in the MR analysis. As genetic variants follow the laws of segregation assortment and independent assortment, MR results are less likely to be influenced by reverse causality and environmental factors that may confound the estimated relationship (20–22). In this study, we conducted two-sample MR analysis to explore the causal relationship between HP infection-related antibodies levels and PD risk and clinical symptoms of PD. Given the overlapping pathogenic mechanisms between secondary parkinsonism and PD, we also examined the association between HP infection and secondary parkinsonism. Additionally, we investigated the reverse causal relationship between PD and HP infection.

## 2 Materials and methods

### 2.1 Mendelian randomization design

**Figure 1:**
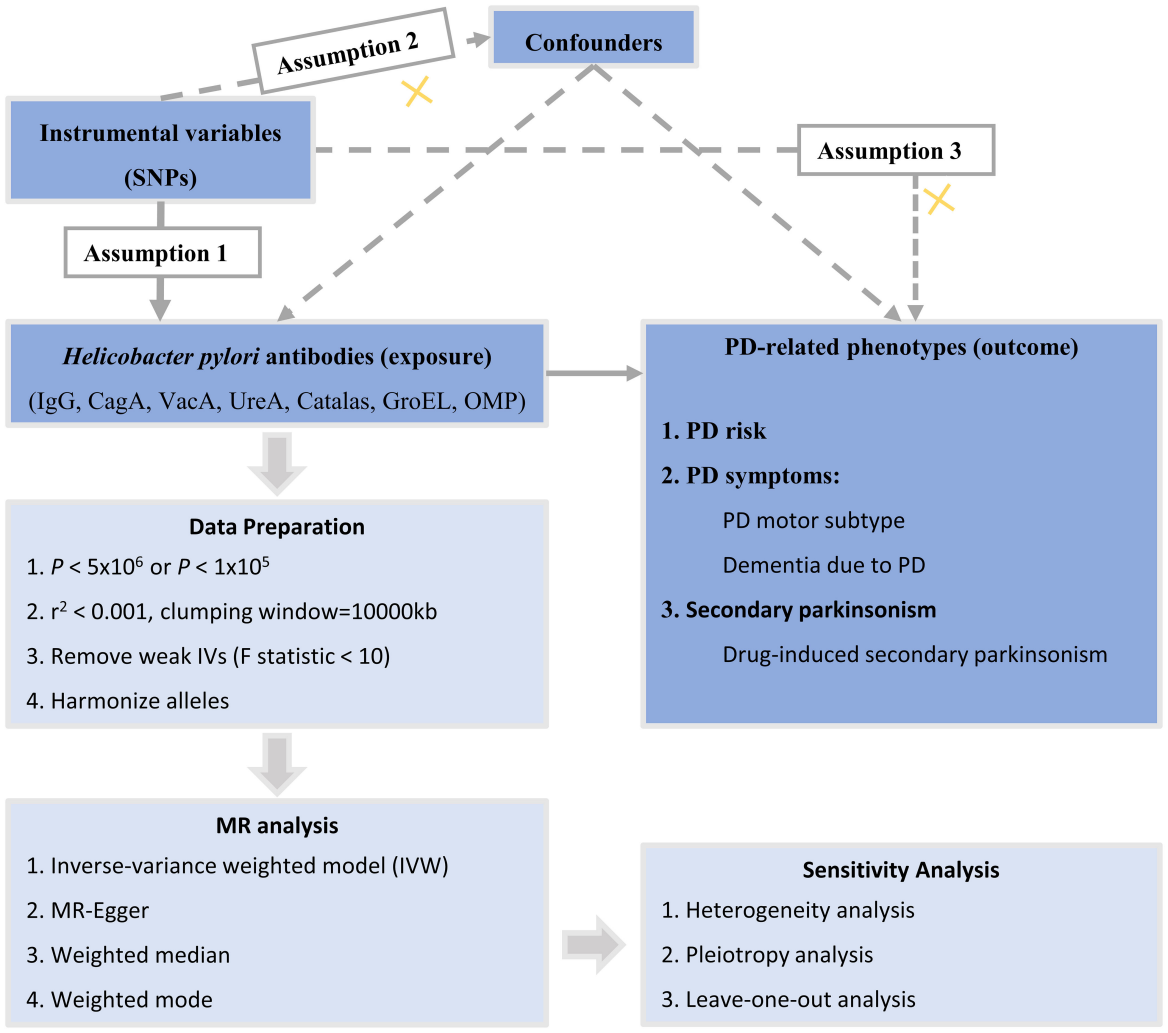
Overall design of the MR study. MR, Mendelian randomization; SNPs, single-nucleotide polymorphisms; PD, Parkinson’s disease; CagA, cytotoxin-associated gene-A; GroEL, chaperonin GroEL; OMP, outer membrane protein; UREA, urease subunit-A; VacA, vacuolating cytotoxin-A.

The study design of this research is illustrated in **Figure 1**. In this current study, we used genetic variants as IVs for MR analysis. The validity of our MR study is based on three core assumptions: (1) the assumption of relevance: genetic variants are highly correlated with the exposure; (2) the assumption of independence: genetic variants are unrelated to confounding factors; and (3) the assumption of exclusion restrictions: genetic variants only affect the outcome through the exposure and not through other pathways.

### 2.2 GWAS data sources

The data used in this study was derived from previously published GWAS. To avoid bias due to population stratification, this study only utilized GWAS summary data from individuals of European ancestry. HP infection was defined based on measurements of serum-specific antibodies against HP proteins. The GWAS dataset for HP protein antibodies was obtained from the UK Biobank and included 8,735 individuals with seven antibody levels for anti-H. pylori IgG, GroEL, OMP, UREA, CagA, VacA, and catalase (9).

We selected five PD-related phenotypes, including PD, PD motor subtype, PD dementia, Secondary parkinsonism, and drug-induced secondary parkinsonism. The summary statistics data for PD were obtained from the largest published meta-analysis of PD GWAS made by International Parkinson’s Disease Genomics Consortium, involving 33,674 PD cases and 449,056 European ancestry controls (23). The summary statistics data for PD motor subtype were derived from multiple North-American and European PD research cohorts, including 3,212 European ancestry PD cases (24). We chose the tremor to postural instability/gait difficulty (PIGD) score ratio as a measure of PD motor subtype because it provides a continuous outcome based on the tremor/PIGD score ratio and has the advantage of a larger sample size (24). The summary statistics data for dementia due to PD, secondary parkinsonism, and drug-induced secondary parkinsonism were obtained from over 21,000 individuals in the Finnish population. All these summary data can be found and downloaded from the IEU OpenGWAS project (https://gwas.mrcieu.ac.uk/). Details of the included GWAS data in this study are provided in **Supplementary Table 1**.

### 2.3 Instrumental variables selection

A series of quality control measures were implemented to select eligible IVs for HP infection that meet the assumptions of MR analysis. In order to achieve more comprehensive results, we applied two threshold levels to filter SNPs related to the exposure. Specifically, a stringent significance threshold (*P* < 5×10^-6^) was used for the primary analysis results, and a lenient significance threshold (*P* < 1×10^-5^) was used for sensitivity analysis (25). We did not select a genome-wide significance threshold (*P* < 5 × 10^-8^) due to insufficient SNPs available for MR analysis. Additionally, the IVs selected show no significant correlation with the PD phenotype (*P* < 5 × 10^-8^). The linkage disequilibrium threshold was set at *R* ^2^ = 0.001, and the distance to search for linkage disequilibrium *R* ^2^-values was set at 10,000 kb. If SNPs selected from the exposure were not present in the outcome dataset, proxy SNPs with significant correlation (*R* ^2^ > 0.8) to the selected variants were used. Subsequently, we evaluated the strength of association between SNPs and the phenotype using the *F*-statistic (*F* = *β* ^2^/se^2^) for each SNP. SNPs with an *F*-statistic below 10 were considered weak IVs and were excluded from the analysis (26). SNPs harmonization was performed to ensure consistency of effect alleles in the exposure and outcome datasets, eliminating ambiguous SNPs with intermediate allele frequencies and those with inconsistent alleles. Detailed information on the IVs is provided in the **Supplementary 2**, **3**. We also conducted reverse MR analysis by treating PD-related phenotypes as the exposure, extracting PD IVs with a significance threshold of *P* < 5×10^-8^, and the remaining phenotypes with a significance threshold of *P* < 5×10^-6^.

### 2.4 Statistical analysis

We conducted two-sample MR analyses to determine the potential causal effects of seven different HP antibodies on PD-related phenotypes. Four methods were used to detect causal effects between the exposure and outcomes, including inverse variance weighted (IVW), MR-Egger, weighted median, and weighted mode method. The IVW technique combines a meta-analysis approach with Wald estimation for each SNP, but it is applicable only when there is no horizontal pleiotropy (27). The IVW method is characterized by its simplicity and higher effectiveness. MR-Egger regression allows for the assessment of pleiotropy using an intercept term. MR-Egger regression assumes that more than 50% of the IVs are affected by horizontal pleiotropy. If the intercept term is zero, the results of MR-Egger regression are consistent with IVW, indicating no horizontal pleiotropy (28). MR-Egger regression provides an estimate that is not affected by violations of the standard IVs assumptions. The weighted median method allows for unbiased causal effect estimation even when up to 50% of the IVs are invalid (29). The weighted median method offers superior precision compared to MR-Egger analysis. The weighted mode calculates the causal effect of the largest cluster of valid IVs. The weighted mode method remains consistent even in the presence of invalid instruments when the highest number of similar individual instrument causal effect estimates is derived from valid instruments (30). These methods are based on different assumptions and conditions, and complement each other. If the results of at least one MR analysis method are significant (*P* < 0.05), we consider a causal relationship between HP antibody levels and the outcomes (31). If the significance results of MR analyses are consistent under different significance thresholds for IVs selection, the results are considered robust.

Multiple sensitivity analyses were conducted to validate the robustness of the MR findings. We used Cochran’s Q statistics to detect heterogeneity through the IVW and MR-Egger methods (32). The presence of heterogeneity among IVs should be taken into account if statistically significant (*P* < 0.05). The presence of horizontal pleiotropy may pose a challenge to the second MR hypothesis. Therefore, two methods were employed to assess potential horizontal pleiotropy. The MR-Egger regression examined whether the results were driven by directional horizontal pleiotropy (28). The intercept derived from the MR-Egger method was employed to evaluate the Instrument Strength Independent of Direct Effect (InSIDE) assumption, which posits that horizontal pleiotropic effects are independent of variant-exposure associations. A *P*_intercept_ < 0.05 indicates the presence of horizontal pleiotropy. The Mendelian randomization pleiotropy residual sum and outlier (MR-PRESSO) detected any outliers reflecting potential pleiotropy bias and corrected for horizontal pleiotropy (33). The number of distributions in the MR-PRESSO analysis was set to 1000. *P* < 0.05 was considered statistically significant. Additionally, to determine whether the causal relationship of the MR analysis was caused by a single SNP (potential heterogeneous SNP), leave-one-out sensitivity analyses were conducted to validate the stability of causal effect estimates. The method involved sequentially excluding each SNP from the IVs to assess the presence of potential outliers.

Finally, a reverse MR analysis was performed between five PD-related phenotypes and HP protein antibodies to examine whether a reverse causal association existed. If the identified significant causal relationship in the forward MR analysis is also significant in the reverse MR analysis, then this relationship will be considered to exhibit reverse causality. The reverse MR procedure was the same as that for the above TSMR analysis.

Statistical analysis was performed using R software (version 4.1.3). The MR analyses were conducted using the “TwoSampleMR” package (34) (version 0.5.10) for MR analysis, and the MR-PRESSO R package (version 1.0) was used for MR-PRESSO.

## 3 Results

Based on a stringent significant threshold of *P* < 5 × 10^−6^, there were 11, 15, 5, 10, 10, 15, and 9 IVs for anti-HP IgG, CagA, GroEL, OMP, UREA, VacA, and catalase antibody levels, respectively. Based on a lenient locus-wide significant threshold of *P* < 1 × 10^−5^, there were 21, 26, 10, 18, 24, 25, and 16 IVs for anti-HP IgG, CagA, GroEL, OMP, UREA, VacA, and catalase antibody levels, respectively. The *F*-statistics for all SNPs were greater than 10, indicating the absence of weak IVs (**Supplementary Tables 2**, **3**).

### 3.1 Causal relationship of *Helicobacter pylori* infection and Parkinson’s disease risk

Under the stringent significant threshold, genetically predicted HP antibody levels, including anti-HP IgG, CagA, GroEL, OMP, UREA, VacA, and catalase antibody, showed no association with the risk of PD using the IVW method [odds ratio (OR), 1.00–1.08; *P* = 0.089–0.999; **Table 1**]. Similar results were obtained when using the MR-Egger, weighted median, and weighted model methods (**Supplementary Table 4**). The non-significant results remained consistent under the lenient significant threshold (**Supplementary Table 5**). Reverse MR analysis revealed no reverse causal relationship between PD risk and HP antibodies levels (**Supplementary Table 6**).

**Table 1:** MR results of causal links between HP antibodies levels on PD (IVW method).

Exposure	Outcome	Nsnp	locus-wide significance	OR (95% CI)	P value
Anti-HP IgG	PD	9	*P* < 5 × 10^-6^	1.00 (0.63-1.58)	0.999
		16	*P* < 1 × 10^-5^	1.08 (0.77-1.52)	0.664
CagA	PD	10	*P* < 5 × 10^-6^	1.00 (0.93-1.07)	0.97
		20	*P* < 1 × 10^-5^	0.99 (0.94-1.04)	0.637
Catalase	PD	9	*P* < 5 × 10^-6^	1.08 (0.99-1.17)	0.089
		16	*P* < 1 × 10^-5^	1.05 (0.98-1.12)	0.183
GroEL	PD	4	*P* < 5 × 10^-6^	1.02 (0.87-1.20)	0.799
		8	*P* < 1 × 10^-5^	1.02 (0.91-1.15)	0.713
OMP	PD	7	*P* < 5 × 10^-6^	1.04 (0.94-1.16)	0.450
		14	*P* < 1 × 10^-5^	1.00 (0.92-1.09)	0.960
UREA	PD	10	*P* < 5 × 10^-6^	1.07 (0.97-1.17)	0.183
		23	*P* < 1 × 10^-5^	1.01 (0.94-1.09)	0.704
VacA	PD	15	*P* < 5 × 10^-6^	1.01 (0.94-1.09)	0.714
		23	*P* < 1 × 10^-5^	1.03 (0.97-1.09)	0.317

MR, Mendelian randomization; HP, Helicobacter pylori; OR, odd ratio; CI, confidence interval; PD, Parkinson’s disease; IVW, inverse-variance weighted method; HP, Helicobacter Pylori; CagA, cytotoxin-associated gene-A; GroEL, chaperonin GroEL; OMP, outer membrane protein; UREA, urease subunit-A; VacA, vacuolating cytotoxin-A.

### 3.2 Causal relationship of *Helicobacter pylori* infection and Parkinson’s disease symptoms

Under the stringent significant threshold (**Table 2** and **Supplementary Table 4**), genetically predicted HP CagA antibody level was negatively associated with the tremor/PIGD score ratio using the IVW method (*β* = -0.162; Standard Error (SE) = 0.05; *P* = 0.002) and the weighted median method (*β* = -0.191; SE = 0.07; *P* = 0.007). Genetically predicted HP GroEL antibody level was also negatively associated with the tremor/PIGD score ratio using the IVW method (*β* = -0.249; SE = 0.10; *P* = 0.010). In contrast, genetically predicted HP OMP antibody level was positively associated with the tremor/PIGD score ratio using the MR-Egger method (*β* = 0.461; SE = 0.18; *P* = 0.048). Additionally, genetically predicted HP VacA antibody was identified as a risk factor for PD dementia using the MR-Egger method (OR = 1.93; 95% Confidence Interval (CI) = 1.10–3.39; *P* = 0.040).

**Table 2:** MR positive results of causal links between HP antibodies levels on PD-related phenotypes (*P* < 5 × 10^-6^).

Exposure	Outcome	Nsnp	Methods	OR (95% CI)	Beta	*P* value	*P* value (Egger intercept)	*P_ivw_* value (Cochran’s Q)	*P* value (MR-PRESSO)
CagA	PD motor subtype (tremor/PIGD score ratio)	10	IVW	0.85 (0.77-0.94)	-0.162	0.002	0.608	0.409	0.814
CagA	PD motor subtype (tremor/PIGD score ratio)	10	Weighted median	0.83 (0.72-0.95)	-0.191	0.007			
GroEL	PD motor subtype (tremor/PIGD score ratio)	10	IVW	0.78 (0.64-0.94)	-0.249	0.010	0.350	0.665	0.409
OMP	PD motor subtype (tremor/PIGD score ratio)	10	MR Egger	1.59 (1.12-2.24)	0.461	0.048	0.058	0.083	0.585
VacA	Dementia due to PD	7	MR Egger	1.93 (1.10-3.39)	0.660	0.040	0.079	0.095	0.040
OMP	Drug-induced secondary parkinsonism	7	IVW	2.08 (1.06-4.08)	0.732	0.033	0.377	0.370	0.563

MR, Mendelian randomization; HP, Helicobacter pylori; OR, odd ratio; CI, confidence interval; PD, Parkinson’s disease; PIGD, postural instability/gait difficulty; IVW, inverse-variance weighted method; CagA, cytotoxin-associated gene-A; GroEL, chaperonin GroEL; OMP, outer membrane protein; VacA, vacuolating cytotoxin-A.

Under the lenient significant threshold (**Table 3** and **Supplementary Table 5**), genetically predicted HP CagA antibody level was negatively associated with the tremor/PIGD score ratio using the IVW method (*β* = -0.083; SE = 0.04; *P* = 0.018) and the weighted mode method (*β* = -0.193; SE = 0.09; *P* = 0.038). Similarly, genetically predicted HP OMP antibody level was positively associated with the tremor/PIGD score ratio using the MR-Egger method (*β* = 0.352; SE = 0.14; *P* = 0.026). Additionally, genetically predicted HP Catalase and VacA antibody were identified as risk factors for PD dementia using the weighted median method (OR = 1.45; 95% CI = 1.01–2.09; *P* = 0.045) and the MR-Egger method (OR = 1.81; 95% CI = 1.05–3.11; *P* = 0.043), respectively.

**Table 3:** MR positive results of causal links between HP antibodies levels on PD-related phenotypes (*P* < 1 × 10^-5^).

Exposure	Outcome	Nsnp	Methods	OR (95% CI)	Beta	*P* value	*P* value (Egger intercept)	*P_ivw_* value (Cochran’s Q)	*P* value (MR-PRESSO)
CagA	PD motor subtype (tremor/PIGD score ratio)	17	IVW	0.92 (0.86-0.99)	-0.083	0.018	0.608	0.409	0.586
CagA	PD motor subtype (tremor/PIGD score ratio)	17	Weighted mode	0.82 (0.70-0.97)	-0.193	0.038			
OMP	PD motor subtype (tremor/PIGD score ratio)	13	MR Egger	1.42 (1.09-1.86)	0.352	0.026	0.058	0.083	0.288
Catalase	Dementia due to PD	15	Weighted median	1.45 (1.01-2.09)	0.374	0.045	0.646	0.254	0.686
VacA	Dementia due to PD	23	MR Egger	1.81 (1.05-3.11)	0.594	0.043	0.079	0.095	0.031
OMP	Drug-induced secondary parkinsonism	15	IVW	1.94 (1.24-3.04)	0.665	0.004	0.377	0.370	0.265

MR, Mendelian randomization; HP, Helicobacter pylori; OR, odd ratio; CI, confidence interval; PD, Parkinson’s disease; PIGD, postural instability/gait difficulty; IVW, inverse-variance weighted method; CagA, cytotoxin-associated gene-A; OMP, outer membrane protein; VacA, vacuolating cytotoxin-A.

**Figure 2:**
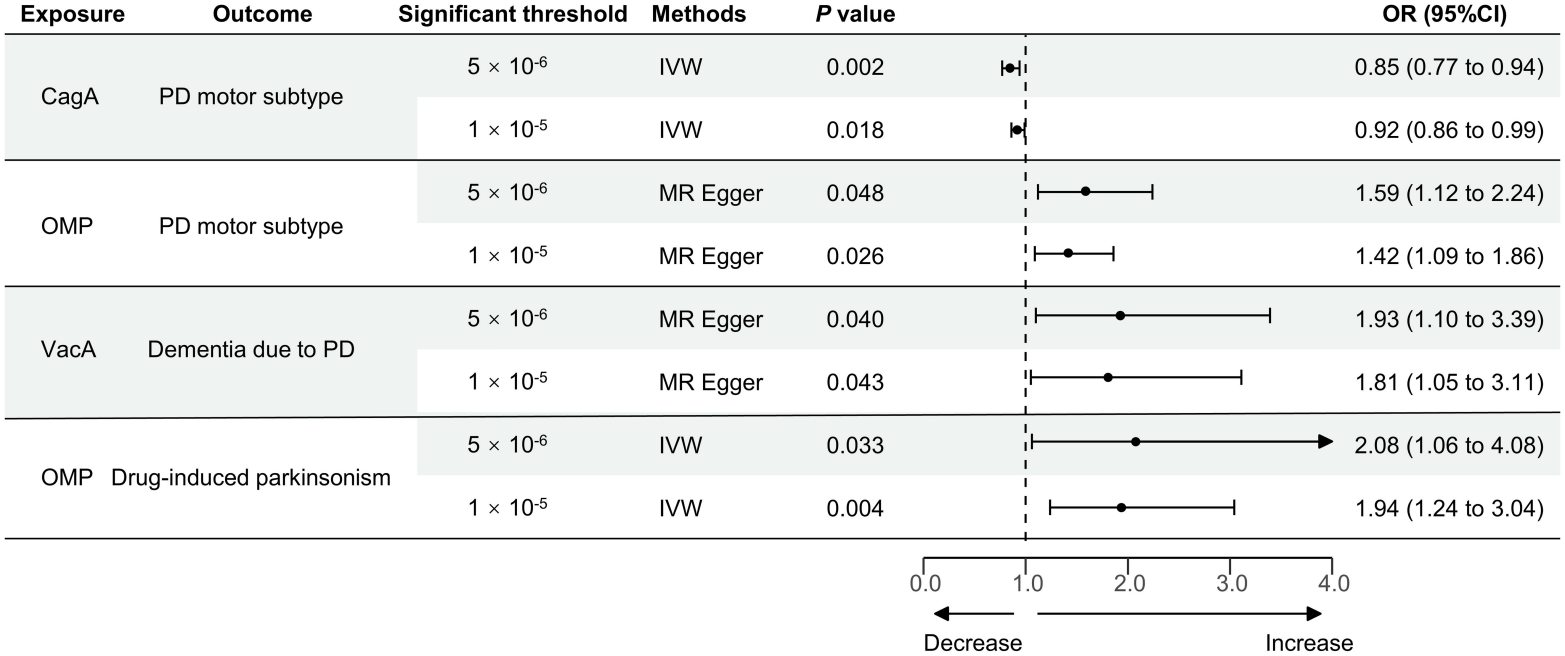
MR stable results of the positive causal effect of HP infection on PD-related phenotypes. MR, Mendelian randomization; HP, Helicobacter pylori; PD, Parkinson’s disease; IVW, inverse-variance weighted method; CagA, cytotoxin-associated gene-A; OMP, outer membrane protein; VacA, vacuolating cytotoxin-A; OR, the odds ratio. 95% CI, 95% confidence interval.

Finally, the causal relationship between HP CagA antibody level and the tremor/PIGD score ratio using the IVW method, HP OMP antibody level and the tremor/PIGD score ratio using the MR Egger method, HP VacA antibody level and dementia due to PD using the MR Egger method, and HP OMP antibody level and drug-induced secondary parkinsonism using the IVW method, all yielded consistent results under both IVs selection thresholds, as shown in **Figure 2**. Furthermore, reverse MR analysis revealed no reverse causal relationships for these positive associations (**Supplementary Table 6**).

### 3.3 Causal relationship of *Helicobacter pylori* infection and secondary parkinsonism

There was no evidence to suggest a causal association between HP antibody levels and secondary parkinsonism. However, under the stringent significant threshold (**Table 2** and **Supplementary Table 4**), genetically predicted HP OMP antibody was identified as a risk factor for drug-induced secondary parkinsonism using the IVW method (OR = 2.08; 95% CI = 1.06–4.08; *P* = 0.033). This result was replicated under lenient conditions (OR = 1.94; 95% CI = 1.24–3.04; *P* = 0.004; **Table 3** and **Supplementary Table 5**). Reverse MR analysis found no evidence of a causal effect of drug-induced secondary parkinsonism on HP antibodies levels (**Supplementary Table 6**).

### 3.4 Sensitivity analysis results

**Figure 3:**
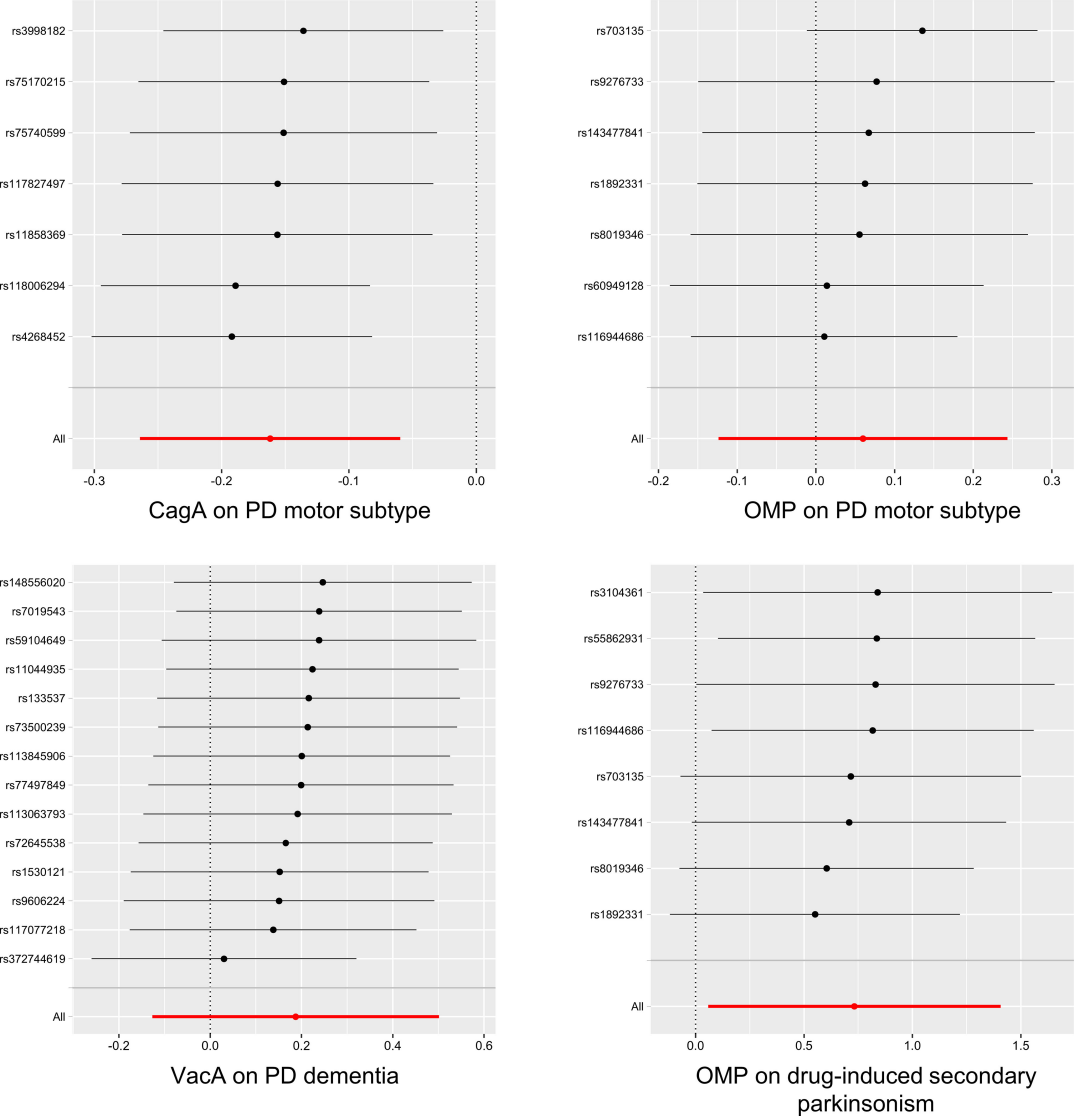
The results of Leave-one-out analysis for HP infection on PD-related phenotypes (*P* < 5×10^-6^). HP, Helicobacter pylori; PD, Parkinson’s disease; CagA, cytotoxin-associated gene-A; OMP, outer membrane protein; VacA, vacuolating cytotoxin-A.

In the sensitivity analyses, heterogeneity analysis showed no evidence of a significant causal effect of HP antibodies levels on the tremor/PIGD score ratio, PD dementia, and drug-induced secondary parkinsonism. Except for a potential pleiotropic effect between HP VacA antibody levels and PD dementia (MR-PRESSO global test *P* = 0.031 at lenient condition and *P* = 0.040 at stringent condition), no other significant causal associations were detected for pleiotropy under the MR Egger intercept test and MR-PRESSO global test (**Tables 2**, **3**). The detailed results of all sensitivity analyses were shown in **Supplementary Tables 7**-**12**. In the Leave-one-out analysis, only two SNPs (rs116944686 and rs145350770) were identified as driving the association between HP OMP antibody levels and the tremor/PIGD score ratio under lenient conditions (**Figure 3** and **Supplementary Figure 1**).

## 4 Discussion

In this study, we utilized publicly available GWAS data and employed the MR analysis method to investigate the causal relationships between seven HP infection-related antibodies and five PD-related phenotypes. Our findings suggest that none of the HP infection-related antibodies have a causal relationship with an increased risk of PD. However, these antibodies are associated with clinical symptoms of PD. Specifically, CagA and OMP antibodies are causally linked to a decreased and increased tremor/PIGD score ratio, respectively, while VacA antibodies increase the risk of PD dementia. Furthermore, we discovered that OMP antibody level is associated with an increased risk of drug-induced secondary parkinsonism. Our study suggests that a nuanced approach to managing HP infection in patients with PD, especially concerning the management of PD symptoms, should be considered. Early detection and appropriate treatment of HP infection could potentially mitigate the progression or exacerbation of PD symptoms.

As early as 1960, researchers identified a potential association between HP infection and PD (35), which has since been supported by numerous observational studies. In our previous study, we reviewed nine meta-analyses and found robust evidence supporting a strong association between HP and PD compared to other microorganisms (14). However, currently, there is no clear evidence to suggest that HP infection directly causes PD or vice versa. Our study indicates that there is no causal relationship between HP infection and PD based on genetic information. We hypothesize that HP infection may be not directly associated with synuclein deposition, which is a core pathogenic mechanism of PD. Therefore, advocating for the use of HP eradication therapy to prevent PD may not be supported based on our findings. The mechanisms by which HP infection may contribute to PD are believed to involve the microbiome-gut-brain axis and the cytotoxin-neuroinflammation hypothesis (8). These hypotheses emphasize that HP may act as a risk factor rather than a causative factor for PD. On one hand, HP infection may disrupt the balance of the gut microbiota through virulence factors (such as CagA, VacA, and Ure), indirectly impacting the brain via the gut-brain axis (36, 37). On the other hand, HP infection may disrupt the blood-brain barrier and induce neuroinflammation through cytotoxins, indirectly affecting neuronal cells (38).

Previous studies link HP infection to PD, not only in terms of comorbidity, but also in relation to clinical symptoms and severity of PD (13–17). Our results demonstrate a causal relationship between HP infection and both motor and cognitive symptoms of PD. Several meta-analyses have shown that PD patients with HP infection increased clinical severity, more severe motor symptoms, and poorer medication response (16, 17). Interventional studies have found that eradicating HP improves motor symptoms, particularly stride length and symptom fluctuations, and enhances clinical response to levodopa in PD patients (15, 39–41). Previous research has indicated that CagA positivity, along with being VacA and urease-B immunoblot negative, increases the predicted probability of being labeled as parkinsonian by the age of 80, serving as a biomarker for the risk and progression rate of idiopathic PD (37). Furthermore, the antibody profile-based discriminant index is also associated with symptoms such as gait, posture, bradykinesia, stoop, and cognition (39). Our results further reveal that different antibody types influence different symptoms of PD. Among them, CagA and OMP antibodies modulate the subtype proportions of motor symptoms. The motor subtypes of PD are primarily classified as tremor-dominant and PIGD subtypes (24). Our findings imply that CagA antibody shows a negative association with the tremor/PIGD score ratio, suggesting a higher tendency towards inducing PIGD. In contrast, OMP antibody exhibits a positive association with the tremor/PIGD score ratio, indicating a stronger association with tremor. Different motor subtypes in PD involve varying mechanisms and simultaneously impact disease progression to different extents (42, 43). For instance, studies have shown that PIGD is associated with increased cognitive impairment and reduced response to levodopa (44). Motor symptoms in PD stem from dopamine dysfunction. Research indicates that CagA-positive HP strains could induce variations in dopamine, serotonin, and other hormone levels in the circulatory system, potentially causing damage across multiple systems, including the central nervous system, and manifesting as associated symptoms (45). A study shows that L-dopa may directly interact with the OMPs of HP responsible for adhesion to gastric epithelial cells. This interaction alters the pharmacokinetics of levodopa and subsequently affects the treatment of motor symptoms (46). Additionally, VacA antibody is associated with an increased risk of cognitive impairment in PD. VacA activates p38MAPK and induces the activation of activating transcription factor 2 (8). The p38MAPK signaling pathway plays a role in neuroinflammatory responses facilitated by microglia and astrocytes. Animal research suggests that the p38MAPK pathway contributes to inflammation triggered by β (1-42) deposition and cholinergic hypofunction (47), and involves with several cognitive impairment disorders (48–50). In summary, our study suggests that after synuclein deposition, HP infection may be involved in the occurrence of different symptoms of PD through various mechanisms. Monitoring different antibody types may help predict patients with different symptom subtypes, and eradicating HP may contribute to modulating and improving different symptoms. For example, for drug-resistant tremor-predominant PD, monitoring circulating CagA antibody levels is necessary, while PD patients presenting cognitive symptoms require monitoring of circulating VacA antibody levels. These patients may benefit from HP eradication. In conducting clinical trials, we suggest simultaneously monitoring the titers of different HP antibodies and PD clinical symptoms to clarify their relationship further. Exploring interactions of different HP antibodies with dopamine pharmacokinetics, gut microbiota (gut-brain axis), or central neuropathology related to PD (synuclein in brain tissues such as the striatum, substantia nigra, etc.) may help elucidate the underlying mechanisms.

Previous studies have mainly explored the relationship between idiopathic Parkinson’s syndrome or PD and HP infection. We investigated for the first time the association between HP infection and secondary parkinsonism and found that OMP antibody level increases the risk of drug-induced secondary parkinsonism. This could be related to HP infection-induced gastrointestinal motility disorders, which affect drug absorption (51). Previous research has shown that HP infection may reduce the bioavailability of levodopa and decrease dopaminergic status (8, 13, 52, 53). Further exploration is needed to determine if patients with HP infection are more susceptible to drug-induced secondary parkinsonism.

This study has certain limitations. Firstly, it should be noted that the sample size for HP infection GWAS data is small (< 1000), which may lead to the omission of important IVs, particularly in the case of CagA, where only 985 individuals were included in the GWAS. Therefore, caution should be exercised when interpreting negative results, and positive results need to be further validated with an expanded sample size. Secondly, HP infection GWAS is based on serological samples, and there is a distinction between HP seropositivity and actual ongoing infection. False-negative or false-positive results cannot be ruled out, so our findings should be interpreted cautiously. Thirdly, the GWAS data for PD motor subtypes is partially sourced from the UK, and we are unable to determine if there is any overlap with the GWAS data for HP infection. Lastly, the dataset used primarily includes European populations, so the results may not be generalizable to other populations, and further exploration is needed in Asian populations.

## 5 Conclusions

In conclusion, this study explores the causal relationship between seven HP infection-related antibodies and five PD-related phenotypes through two-sample MR analysis. Our findings suggest that none of the HP infection-related antibodies have a causal relationship with an increased risk of PD, but they may affect the motor and cognitive symptoms of PD and increase the risk of drug-induced secondary parkinsonism. Our research supports the improvement of clinical symptoms of PD through HP eradication.

## Data availability statement

The original contributions presented in the study are included in the article/**Supplementary Material**. Further inquiries can be directed to the corresponding author.

## Author contributions

XW: Conceptualization, Data curation, Formal analysis, Investigation, Methodology, Project administration, Supervision, Validation, Writing – original draft, Writing – review & editing. DJ: Conceptualization, Data curation, Formal analysis, Investigation, Methodology, Project administration, Software, Writing – original draft, Writing – review & editing. XZ: Data curation, Formal analysis, Validation, Writing – review & editing. RW: Data curation, Validation, Writing – review & editing. FY: Data curation, Writing – review & editing. CX: Conceptualization, Data curation, Formal analysis, Investigation, Project administration, Resources, Supervision, Validation, Writing – original draft, Writing – review & editing.
