# Supplementary material for: Causal associations between Helicobacter Pylori infection and the risk and symptoms of Parkinson’s disease: a Mendelian randomization study
Source: Front Immunol. 2024 Aug 6;15:1412157. doi: 10.3389/fimmu.2024.1412157 (PMC11333313; doi:10.3389/fimmu.2024.1412157)
Supplement: Supplementary file 1 [file DataSheet_1.docx]

Supplementary Material

# Supplementary Figures


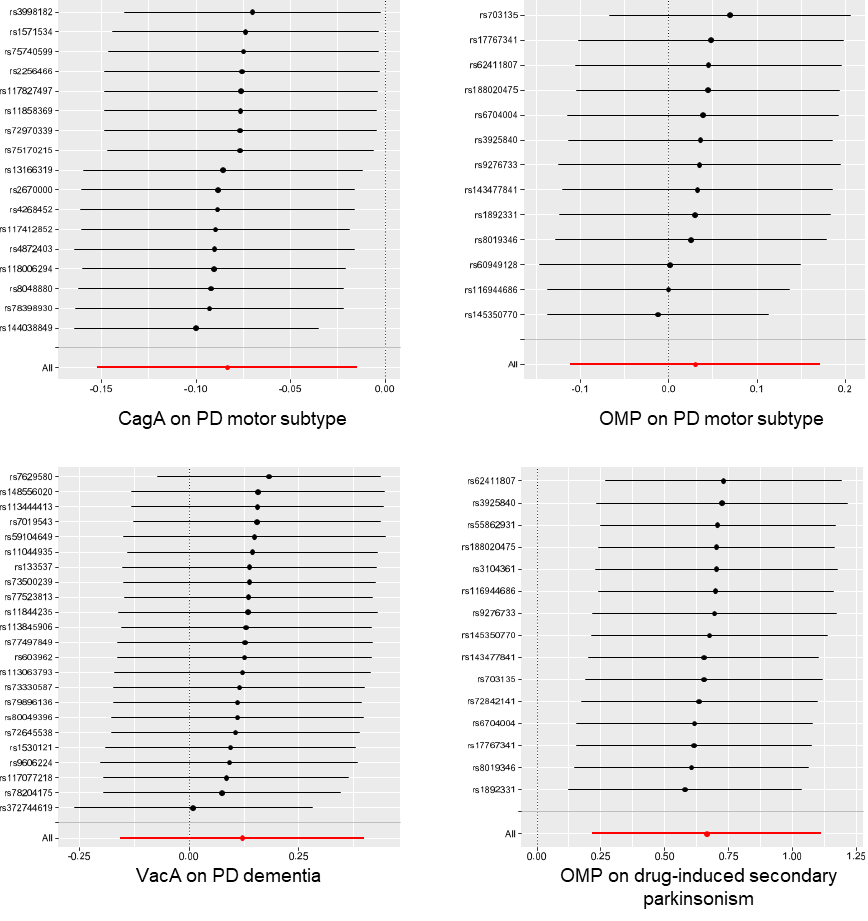


**Supplementary Figure 1.** **The results of Leave-one-out analysis for Helicobacter Pylori infection on Parkinson's disease-related phenotypes (P < 1×10^-5^).** PD, Parkinson's disease; CagA, cytotoxin-associated gene-A; OMP, outer membrane protein; VacA, vacuolating cytotoxin-A.
